# Mutations Increasing Cofactor Affinity, Improve Stability and Activity of a Baeyer–Villiger Monooxygenase

**DOI:** 10.1021/acscatal.2c03225

**Published:** 2022-09-13

**Authors:** Hamid R. Mansouri, Oriol Gracia Carmona, Julia Jodlbauer, Lorenz Schweiger, Michael J. Fink, Erik Breslmayr, Christophe Laurent, Saima Feroz, Leticia C. P. Goncalves, Daniela V. Rial, Marko D. Mihovilovic, Andreas S. Bommarius, Roland Ludwig, Chris Oostenbrink, Florian Rudroff

**Affiliations:** † Institute of Applied Synthetic Chemistry, TU Wien, Getreidemarkt 9, 1060 Vienna, Austria; ‡ Biocatalysis and Biosensing Laboratory, Department of Food Science and Technology, BOKU−University of Natural Resources and Life Sciences, Vienna, Muthgasse 18, 1190 Vienna, Austria; § School of Chemical & Biomolecular Engineering, Engineered Biosystems Building (EBB), Georgia Institute of Technology, 950 Atlantic Drive, N.W., Atlanta, Georgia 30332, United States; ∥ Institute of Molecular Modeling and Simulation, University of Natural Resources and Life Sciences, 1190 Vienna, Austria; ⊥ Institut de Chimie de Nice CRNS UMR7272, Université Côte d’Azur, 28 Avenue Valrose, 06108 Nice, France; # Área Biología Molecular, Departamento de Ciencias Biológicas, Facultad de Ciencias Bioquímicas y Farmacéuticas, Universidad Nacional de Rosario (UNR), and Consejo Nacional de Investigaciones Científicas y Técnicas (CONICET), Suipacha 531, S2002LRK Rosario, Argentina; ∞ Department of Biosciences, College of Science, University of Hafr Al Batin, PO Box 1803, Hafr Al Batin, 39524, Saudi Arabia

**Keywords:** protein engineering, enzyme stabilization, cyclohexanone monooxygenase, structure-guided consensus approach, oxidation, mutagenesis

## Abstract

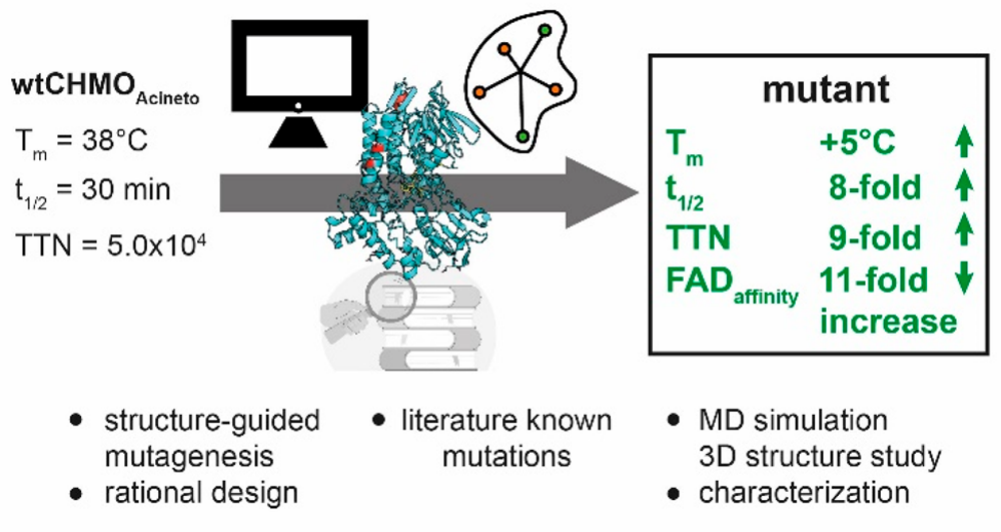

The typically low thermodynamic and kinetic stability of enzymes is a bottleneck for their application in industrial synthesis. Baeyer–Villiger monooxygenases, which oxidize ketones to lactones using aerial oxygen, among other activities, suffer particularly from these instabilities. Previous efforts in protein engineering have increased thermodynamic stability but at the price of decreased activity. Here, we solved this trade-off by introducing mutations in a cyclohexanone monooxygenase from *Acinetobacter* sp., guided by a combination of rational and structure-guided consensus approaches. We developed variants with improved activity (1.5- to 2.5-fold) and increased thermodynamic (+5 °C *T*_m_) and kinetic stability (8-fold). Our analysis revealed a crucial position in the cofactor binding domain, responsible for an 11-fold increase in affinity to the flavin cofactor, and explained using MD simulations. This gain in affinity was compatible with other mutations. While our study focused on a particular model enzyme, previous studies indicate that these findings are plausibly applicable to other BVMOs, and possibly to other flavin-dependent monooxygenases. These new design principles can inform the development of industrially robust, flavin-dependent biocatalysts for various oxidations.

The need and demand for more sustainable methods of producing bulk and fine chemicals derived from renewable resources have been significant driving forces in biocatalysis. Lately, many industrially relevant processes, including enzymes as catalysts, have been established.^1^ Proteins often outcompete chemical counterparts by their chemo-, regio-, and enantioselectivity.^2^ Despite these advantages, they often show reduced stability^3^ in the presence of organic solvents, high substrate or product concentration, and elevated temperatures. The ability to stabilize proteins means manipulating the physicochemical properties to obtain a thermodynamically stable scaffold.^4,5^ One hurdle is to improve thermodynamic stability but not to lose its activity or selectivity. Many efforts have been made to tackle this challenge, but there is no one-size-fits-all strategy. Known strategies are directed evolution^6,7^ and sequence-based phylogenetic analysis to identify thermostable analogous and structure-guided site-directed mutagenesis.^8,9^ Increased protein stability will result in lower process costs per unit of product and make biocatalytic transformations a real alternative to chemical processes.

A prominent example of the enzyme outperforming conventional chemical catalysis is the Baeyer–Villiger oxidation.^10−12^ In chemical synthesis, ketones are oxidized to the corresponding esters or lactones by peracids or peroxides. These strong oxidants are often explosive, are sometimes toxic, are needed in stoichiometric amounts, often do not tolerate other functional groups, and are not as stereoselective as enzymes.^13,14^ A “greener” and a catalytic alternative is the use of Baeyer–Villiger monooxygenases (BVMOs).^15^ These enzymes require nicotinamide and flavin cofactors (nicotinamide adenine dinucleotide, NADPH, and flavin adenine dinucleotide, FAD) and aerial oxygen to perform Baeyer–Villiger oxidations. BVMOs are outperforming traditional catalysis due to their excellent chemo-, regio-, and enantioselectivity.^16,17^ BVMOs are highly valued for their synthetic potential, but their poor stability under process conditions has so far prevented their industrial applications.^18^ BVMOs often get inactivated within a few minutes at elevated temperatures and in the presence of organic solvents.

A few stable BVMOs are known from the literature: phenylacetone monooxygenase (PAMO), thermostable cyclohexanone monooxygenase (TmCHMO), and polycyclic ketone monooxygenase (PockeMO).^19,20^ Although their stability is high, they have either poor enantio-/regioselectivity, limited substrate scope (PAMO),^21^ or low catalytic efficiency (TmCHMO, PockeMO) in contrast to others (e.g., cyclohexanone monooxygenase from *Acinetobacter* sp. NCIMB 9871, CHMO_Acineto_).^18−20,22−24^ Thus, there is an unsolved problem in engineering highly stable and active BVMOs for industrial applications.

We chose CHMO_Acineto_ as the model catalyst for applying our approach to stabilization. CHMO_Acineto_ accepts a wide range of substrates, has high selectivity for Baeyer–Villiger oxidations,^25^ and is efficient.^18,26^ Although the catalyzed activity and selectivity of this enzyme would be valuable for industrial applications,^16,27−30^ its insufficient stability remains uncured.^22,31^

Several studies have used protein engineering to address this limitation: Schmidt et al. and Van Beek et al. introduced disulfide bridges,^32,33^ resulting in an improvement of the transition midpoint temperature (Δ*T*_50_) of thermal denaturation by 5 and 6 °C, respectively. Opperman et al. substituted amino acids susceptible to oxidation, resulting in an increase in *T*_50_ by 7 °C.^34^ Despite the improved stability, all variants had lower activity than the wild-type (WT) enzyme. Engel et al. indicated that none of these mutants could outperform the wild-type enzyme in the production of ε-caprolactone,^35,36^ an important polymer building block for the synthesis of polyesters, thermoplastic polyurethanes, acrylic resins, printing inks, plasticizers, and precursor to nylon-6.^37,38^ Previous thermostability engineering efforts have not yet achieved the desired outcomes of high stability and activity.

We designed our engineering strategy using a combination of (i) a rational-design approach that increases the affinity to the FAD cofactor, (ii) applying a structure-guided consensus approach that compares 31 sequences (motif, FxGxxxHxxxW; x = any canonical amino acid) of BVMOs,^8,9^ and (iii) including “hot-spots” in the sequence that are known for improving stability and activity of CHMO_Acineto_.^39^

This leads to three generations of mutant libraries (L_1_–L_3_, Figure 1). The variants were always fully characterized in their activity, selectivity, and catalytic efficiency, as well as their thermodynamic (*T*_m_) and kinetic stability (*t*_1/2_).

**Figure 1:**
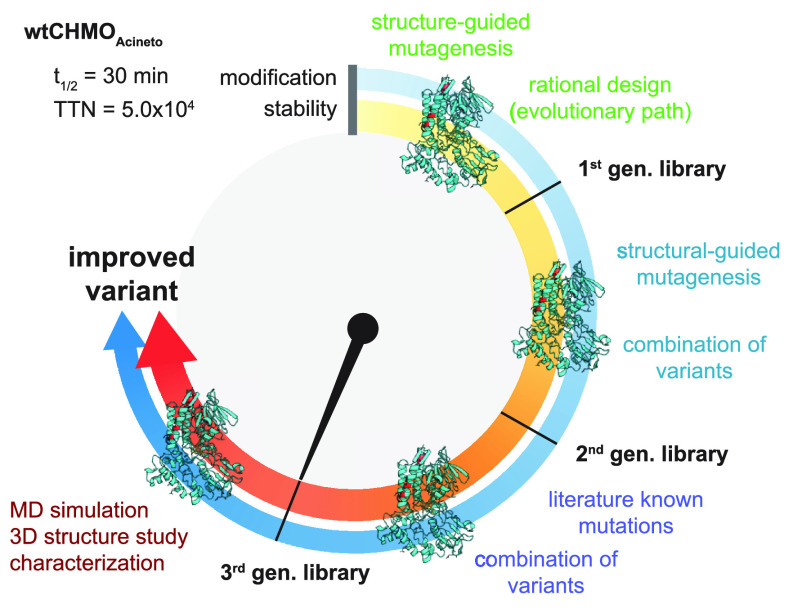
Overview of the individual steps followed in the CHMO_Acineto_ stabilization workflow. Three generations of libraries created with 17 individual variants. Mutations are labeled in red.

First (L_1_), we addressed the low affinity for the cofactor FAD, which we had determined at *K*_d_ = 1–3 μM in an earlier study.^26^ For catalytic activity, it is required to be noncovalently bound to the apoenzyme via the so-called Rossmann fold, a binding domain located at the N-terminus of the enzyme (Figure S1). We had shown that addition of FAD increased the kinetic stability of the wild-type up to 7-fold and that this effect was synergistic with improvements caused by other additives.^26^ No previous study, including ours, has investigated the effect of mutations in the Rossman fold on the binding affinity to FAD.

Following up on this finding, we hypothesized that increased affinity to FAD achieved by protein engineering might also contribute positively to enzyme stability. We studied multiple sequences of BVMOs, specifically their Rossmann fold (Figure S2). We found that BVMOs with high stability (e.g., TmCHMO, PAMO) carry an alanine at position 14 (the second position in the Rossmann fold). CHMO_Acineto_ has a glycine at that position. This finding prompted us to choose G14A as the first mutation (L_1_-1). We measured the thermodynamic and kinetic stability and the activity using differential scanning fluorimetry (DSF) and an NADPH-depletion assay, respectively,^26^ of CHMO-L_1_-1 (Figure 2, Table S3): activity increased by 13%, thermodynamic stability by 1.4 °C (quantified as *T*_m_), and kinetic stability by 30% over the wild-type.

**Figure 2:**
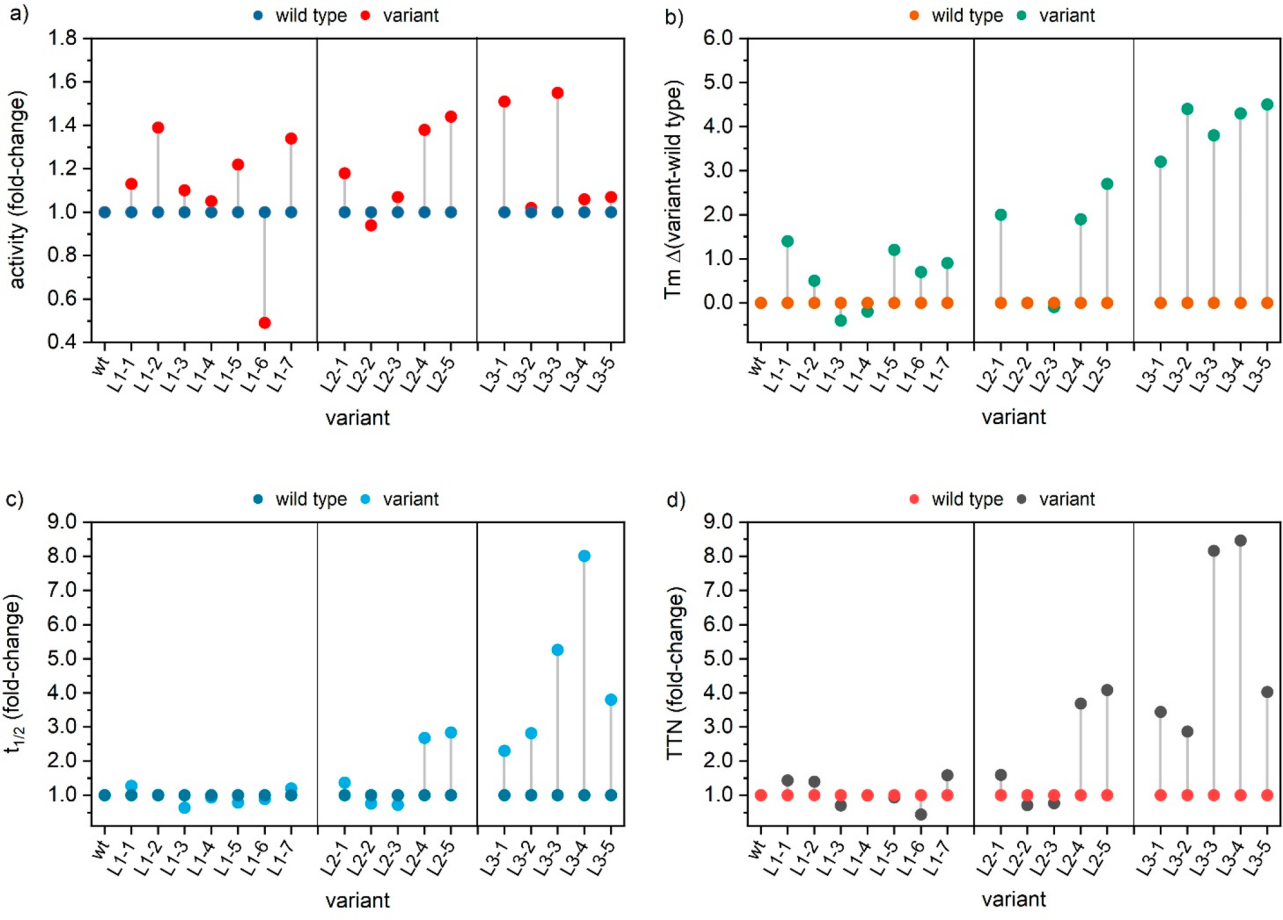
Characterization of best variants. (a) Enzyme activity was measured by monitoring the decrease of NADPH absorbance at 340 nm. Standard assays contained the enzyme (0.05 μM), cyclohexanone (0.5 mM), and NADPH (100 μM) in 50 mM TrisHCl pH 8.5. CHMO_wt_ = 16.4 ± 1.1 U mg^–1^. (b) Thermodynamic stability measured by nanodifferential scanning fluorimetry (nanoDSF): 50 mM TrisHCl, 10 μM FAD, 2 mg mL^–1^ enzyme. CHMO_wt_ = 38.2 °C. (c) Kinetic stability of 1 μM isolated CHMO_Acineto_ at 30 °C in 50 mM TrisHCl buffer, pH 8.5. CHMOwt = 34.4 ± 4.6 min. (d) Total turnover number (TTN) values were obtained from the exponential fit of catalytic enzyme activity under turnover conditions, CHMO_wt_ = 5.04 × 10^4^.^43^

The binding affinity of the G14A variant to FAD was ∼8-fold tighter than in the wild-type (Table S4), determined using our statistically reliable deflavination–titration assay reported previously.^31^ These results supported our hypothesis that position 14 is a critical residue for stability and that stability and activity can be increased simultaneously. High kinetic stability and specific activity (from initial rate measurements) are good predictors for a high total turnover number (“activity”), individually or combined. Our experiments were not designed to distinguish between these cases. Given the large difference between the relative time scales of deactivation (half-life >0.5 h) and of the measurements to determine specific activity (seconds to minutes), it is unlikely that increases in kinetic stability would significantly confound these measurements. With less stable enzymes, that is indeed a problem, as we have reported earlier.^31^

We decided to explore the potential of position 14 and created mutants with the remaining canonical amino acids except methionine, valine, and serine, which failed in the PCR experiments. All variants except G14A and G14R had low or undetectable activity (Table S5). The thermodynamic stability of the additional variants was also much lower than WT (Table S5, Figure S6). These results are compatible with the evolutionary analysis of BVMOs, where only glycine and alanine are found in position 14 (Figure S2).^40−42^ The introduction of arginine did not abolish activity completely (30% of CHMO_Acineto_) while creating a thermodynamically stable variant (+0.3 °C over CHMO_wt_). By inference from the poor or undetectable activity, it is likely that mutations at G14 other than A or W strongly decrease the affinity to FAD, but we cannot exclude other reasons for the lack of activity based on our data.

Next, we envisioned further stabilization by a structure-guided consensus approach, a data-driven method that utilizes structural information and the function of the desired enzyme. It operates on the assumption that the prevalence of amino acids (per position in the sequence) correlates positively with the stability of the protein; a consensus residue will be more stable than the nonconserved amino acids. This method is usually applied to a small family of sequences with low homology, such as BVMOs (Figure 1). All mutations are listed in Table S2.

For the creation of the consensus variants, only positions more than 6 Å away from the active site were allowed in the design (based on a homology model, Figure S3) to not directly interfere with the enzyme activity or affinity for the substrates. Seven mutants were selected and created by these principles, which included the previously tested variant L_1_-1 (G14A). We found the greatest improvements in activity with substitution N336E (40% higher activity than CHMO_wt_). The highest thermodynamic stability came from the previously characterized G14A (*T*_m_ + 1.4 °C over WT) and L_1_-7 (+0.9 °C). Kinetic stability of the latter two increased by 30% and 20% over WT. See Figure 2 and Table S3 for details.

We designed the second-generation library of mutants by combining the best variants from the first generation (Table S2), which led to a further improvement in stability (up to 3 °C higher *T*_m_ and 3-fold the half-life of the WT) and activity (40% increase over WT; Figure 2). Mutations shared among the successful combinations included G14A, N336E, Q451K, T453A, V454E, Q473R, and N477E.

We created a third library consisting of five variants that combined the best mutations of the second generation with three literature-known mutations that increase stability (Table S2): (i) a pair of mutations (T415C/A463C) that were shown to have a beneficial effect on kinetic stability (3-fold half-life);^28^ and (ii) a replacement of oxygen-sensitive methionine by isoleucine (M400I), which had been designed to reduce the rate of unfolding.^34^

In general, all variants of the third generation were more stable (thermodynamically and kinetically) and equally or more active than the wild-type (Table S3)

The best candidates from L_3_ were 51–55% more active and had +4 °C higher thermodynamic stability than CHMO_wt_; their half-life was 5–8-fold that of the WT. These improvements were the highest in this study and are outstanding in the field of BVMOs. To quantify the combined effect of improvements in stability and activity, we estimated the total turnover number of the new mutants (Figure 2, Table S3), which were up to 8-fold the value of the WT.

We tested if the mutations had changed the enantioselectivity or substrate scope using a selection of six variants from libraries L_2_ and L_3_ and five substituted cyclohexanone and cyclobutanones (Figure S7, Table S6). No significant differences of the variants to the WT were found. The substrate screening was performed by whole-cell biocatalysis in the presence of the desired ketone. Conversions were analyzed via GC measurements to rule out activities based on uncoupling reactions.^44^ We also determined the Michaelis–Menten parameters for two variants [L_1_-1 (G14A, improved FAD binding)] and L_3_-4 best variant in stability (G14A, N336E, M400I, Q451K, Y452Y, T453A, V454E, Q473R, and N477E), showing an increased affinity for the substrate and a higher turnover rate in the mutants (Table 1, Table S4).

**Table 1:** Characterization of Michaelis–Menten Kinetics of Top Variants

variant	*K*_m_^a^ (μM)	*k*_cat_^a^ (s^–1^)	*k*_cat_/*K*_m_ (mM^–1^ s^–1^)	*K*_d_^b^ (μM)
CHMO_Acineto_	6.7 ± 2.0	15.0 ± 1.3	2200	1.60 ± 0.06
L_1_-1	3.5 ± 0.3	24.2 ± 1.4	7058	0.19 ± 0.07
L_3_-4	5.0 ± 1.5	16.3 ± 1.8	3187	0.14 ± 0.02

a Catalytic rates were obtained via incubation of the isolated enzyme with varying amounts of substrate, and kinetic parameters (*K*_m_, *k*_cat_) were determined by fitting to the Michaelis–Menten equation.

b The *K*_d_ value was determined by fitting the data of catalytic activity of the holoenzyme versus concentration of FAD.

To confirm whether our hypothesis for library L_1_ (tight binding of FAD increases stability) would still hold true in L_3_, we chose to measure the binding affinity of FAD for the two mutants characterized above, using our statistically reliable assay.^31^ We found that binding to the cofactor was tighter than in the WT by approximately 1 order of magnitude in both variants (Table 1, Table S4), supporting the hypothesis, and not significantly different between the two. While we cannot determine whether G14A is the only mutation that causes an increase in affinity, we can conclude that the effect of G14A is not significantly perturbed by the combination of the other seven mutations in L_3_-4 (N336E, M400I, Q451K, T453A, V454E, Q473R, and N477E). Whether that is the result of insignificant participation or mutual canceling (to a mean value that is insignificantly different from G14A) was not a goal of our experimental design.

We used molecular dynamics to elucidate what structural changes caused by the mutations in most stable variants were responsible for the higher affinity to FAD. Simulations (50 ns, 5 replicate calculations; Figure 3) were performed on a homology model of CHMO_wt_, including the following variants: L_1_-1 (G14A), G14R (the other active variant from the G14 library), and G14T (an inactive variant from that library).

**Figure 3:**
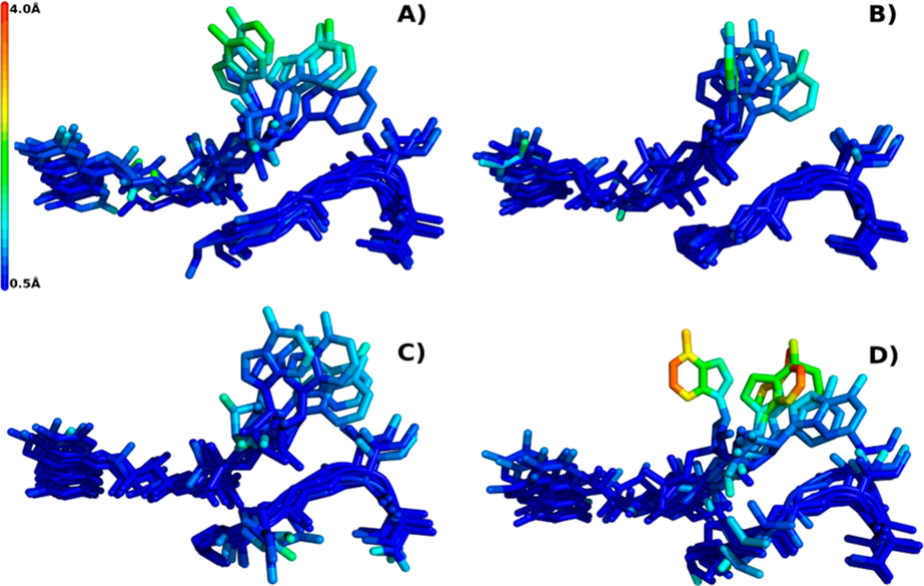
3D representation of the average position of the mutated loop (bottom) and the FAD cofactor (top) for the (A) CHMO_wt_, (B) G14A mutant, (C) G14R mutant, and (D) G14T mutant. The root-mean-square fluctuations are represented in a color gradient: blue (small fluctuations), red (higher fluctuations). Each of the panels shows a superposition of the five MD simulations.

The simulations predicted no significant differences in the backbone or orientations of side chains on the mutated region in any of the variants. The predicted fluctuations for the mutated region were small (∼0.6 Å) and not significantly different between variants, suggesting that it is static.

For the WT and the variant G14T, the simulations positioned the adenosine of FAD detached from its original position and predicted significantly larger fluctuations than for the variants G14A and G14R (Figure 3). This result indicates that the holoenzyme G14A-FAD and G14R-FAD are more rigid than the WT or G14T.

Our structural analysis suggests that the side chain of residue 14 is in close proximity to the FAD cofactor but pointing away from it. We conclude that there is no direct interaction between the side chain and FAD and speculate that the observed stabilization might be achieved by a more complex interaction with the adjacent amino acids. This explanation is compatible with our finding that two drastically different amino acids (alanine and arginine) both lead to an increase in enzymatic activity.

We increased the thermodynamic- (+5 °C *T*_m_), and kinetic stability (8-fold half-life), and the activity (1.5–2.5-fold) of CHMO_Acineto_ by introducing mutations designed with a combination of a rational and structure-guided consensus principles. The changes to the structure had no measurable effect on substrate scope or regio- and enantioselectivity. A single mutation introduced by our design increased the affinity toward the cofactor FAD by ∼11-fold—an increase that was compatible with other mutations introduced later. Previous studies, by ourselves and by others, did not evaluate the influence of mutations on the affinity to the flavin cofactor. Our results show that the model BVMO cyclohexanone monooxygenase from *Acinetobacter* can be stabilized while preserving its catalytic activity and substrate promiscuity. Based on general knowledge in the field, it is plausible that similar improvements could be achieved by this design with closely related BVMOs, and potentially also with other flavin-dependent oxygenases. Many industrially relevant oxidations are catalyzed by these and other enzymes and would benefit from new principles to develop catalysts with high operational stability.

## Supporting Information Available

Additional experimental data as well as detailed experimental procedures (PDF)
