# Supplementary material for: Mutations Increasing Cofactor Affinity, Improve Stability and Activity of a Baeyer–Villiger Monooxygenase
Source: ACS Catal. 2022 Sep 13;12(19):11761–6. doi: 10.1021/acscatal.2c03225 (PMC9552169; doi:10.1021/acscatal.2c03225)
Supplement: Supplementary file 1 — cs2c03225_si_001.pdf [file cs2c03225_si_001.pdf]

# Mutations increasing cofactor affinity improve stability and activity of a Baeyer-Villiger monooxygenase

Hamid R. Mansouri[1], Oriol Gracia Carmona[4], Julia Jodlbauer[1], Lorenz Schweiger[2], Michael J. Fink[1], Erik Breslmayr[2], Christophe Laurent[2], Saima Feroz[1], Leticia C. P. Goncalves, Daniela V. Rial[6], Marko D. Mihovilovic[1], Andreas S. Bommarius[3], Roland Ludwig[2], Chris Oostenbrink[4*], Florian Rudroff[1*]

[1]Institute of Applied Synthetic Chemistry, TU Wien, Getreidemarkt 9, 1060, Vienna, Austria

[2]Biocatalysis and Biosensing Laboratory, Department of Food Science and Technology, BOKU–University of Natural Resources and Life Sciences, Vienna, Muthgasse 18, 1190, Vienna, Austria

[3]School of Chemical & Biomolecular Engineering, Engineered Biosystems Building (EBB), Georgia Institute of Technology, 950 Atlantic Drive, N.W., Atlanta, GA 30332 (USA)

[4]Institute of Molecular Modeling and Simulation, University of Natural Resources and Life Sciences, 1190 Vienna, Austria

[5]Institut de Chimie de Nice CRNS UMR7272, Université Côte d'Azur, 28 Avenue Valrose, 06108 Nice, France

[6]Área Biología Molecular, Departamento de Ciencias Biológicas, Facultad de Ciencias Bioquímicas y Farmacéuticas, Universidad Nacional de Rosario (UNR), and Consejo Nacional de Investigaciones Científicas y Técnicas (CONICET), Suipacha 531, S2002LRK, Rosario, Argentina

**Corresponding authors**: Florian Rudroff, florian.rudroff@tuwien.ac.at; Chris Oostenbrink, chris.oostenbrink@boku.ac.at

## Table of content

A Supporting results 2
- A I Comparison of $CHMO_{Acineto}$ variants ... 2
- A II Rossmann fold sequence in BVMOs ... 2
- A III Prediction of mutants by structure-guided consensus approach ... 3
- A IV Homology model of all three generations of $CHMO_{Acineto}$ ... 4
- A V Library of mutants ... 5
- A VI Characterization of variants ... 6
  - A VI.1 Evaluation of melting temperature ($T_m$) ... 7
- A VII Characterization of site saturated G14 position ... 9
  - A VII.1 Circular dichroism (CD) spectroscopy ... 10
- A VIII Substrate profile ... 12
- A IX MD simulation and structure analysis ... 13

B Materials and Methods 16
- B I Plasmid map and cloning plan ... 16
- B II Site saturation mutagenesis ... 16
- B III Enzyme expression and purification ... 17
- B IV Apoenzyme preparation (Deflavination) ... 17
- B V Enzyme activity and stability measurements ... 18
- B VI Kinetic measurements ... 18
- B VII Melting temperature ($T_m$) ... 18
- B VIII Biotransformation ... 19

C References 20

# A Supporting results

## A I Comparison of $CHMO_{Acineto}$ variants

**Table S1.** $CHMO_{Acineto}$ stability and comparison with few variants.

| Enzyme | $T_m$ | $t_{1/2}$ | $T_{50}$ |
|---|---|---|---|
| **$CHMO_{Acineto}$** | 37-39[a]/31.6[b] | 4min[c]/1.82h[d]/57h[f]/ 17h[d]/11h[c]/17h[e] | 40.5/32.5 |
| **$CHMO_{Acineto}$ Mut15** | | | 47.3 |
| **$CHMO_{Acineto}$ Mut16** | | | 43.4 |
| **$CHMO_{Acineto}$ A255C/A293C** | 40.5[a] | | |
| **$CHMO_{Acineto}$ A325C/L483C** | 40.5[a] | | |
| **$CHMO_{Acineto}$ L323C/A325C** | 44[a] | 45min[c] | |
| **$CHMO_{Acineto}$ T415C/A463C** | 36.5[a]/36.4[b] | 5.2[d] | 38 |
| **$CHMO_{Acineto}$ T415C** | 37.5 [b] | 14.6[d] | 35 |

[a]determined by Thermo-FAD method.
[b]determined by CD-spectroscopy.
[c]measured after incubation at 30 °C.
[d]measured after incubation at 25 °C.
[e]measured after incubation at 52 °C.
[f]measured after incubation at 20 °C

## A II Rossmann fold sequence in BVMOs

The Rossmann fold is a super secondary structure seen in proteins binding nucleotides. It is composed of beta sheets and alpha-helical sections. The alpha helices surround both faces of the sheet to produce a three-layered sandwich, and the beta-strands are hydrogen-bonded to each other forming an extended beta-sheet. The main function of this motif in enzymes is the binding of nucleotide cofactors and/or substrates. The first, third, and sixth position in the Rossman fold is highly conserved and susceptible to changes. In $CHMO_{Acineto}$, the Rossmann fold sequence is composed of -GGGFGG- and is crucial for FAD-binding (Figure S1).

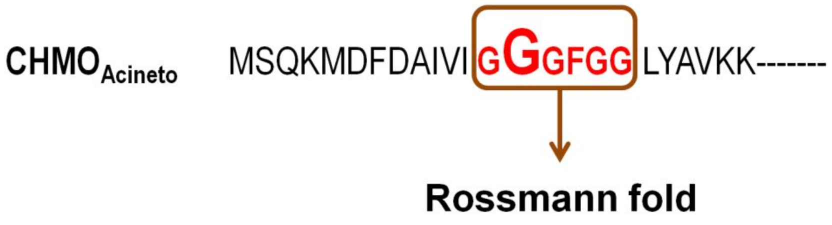


**Figure S1.** Rossmann fold sequence in the $CHMO_{Acineto}$ sequence.

This motif is also highly conserved within the BVMO family. Interestingly in more thermostable variants, a glycine substitution into an alanine is observed. This is especially true for TmCHMO and PAMO (Figure S2).

```
HAPAMO    TAEEDLRAPRWHKDHVASGRDFKVVIIGAGESGMIAALRFK-QAGVPFVIYEKGNDVGGT    179
CPDMO     ---------------DPVVRETDVFIIGGGFGGLLAAVRLQQAGVSDYVMVERAGDYGGT    111
CDMO      ---------------EPKLDHVTFAFIGGGFSGLVTAARLRESGVESVRIIDKAGDFGGV    95
CPMO      ---------------NSVDDTLDVLLIGAGFTGLYQLHHLR-KLGFKVHLVDAGADVGGI    56
PAMO      ---------------RQPPEEVDVLVVGAGFSGLYALYRLR-ELGRSVHVIETAGDVGGV    54
CAMO      ---------------IPEVLNVDALVVGAGVAGIYSTYRLS-RAGLNVQCIDTAGDVGGT    47
CHMO      ---------------MSQKMDFDAIVIGGGFGGLYAVKKLRDELELKVQAFDKATDVAGT    45
TmCHMO    ---------------TTQTPDLDAIVIGAGFGGIYMLHKLRNDLGLSVRVFEKGGGVGGT    47
                                   .:*.*  *:    ::          : . . .*
```

**Figure S2:** Multiple sequence alignment of different BVMOs. Multiple sequence alignment has been performed by MUSCLE (Multiple Sequence Comparison by Log-Expectation).[1]

## A III Prediction of mutants by structure-guided consensus approach

With the assumptions that primordial Earth was hotter than today's climate and thus functional proteins had, on average, to be more stable and that encoding stability in the amino acid sequence follows a Boltzmann-like or similar statistics, the consensus amino acid at each position contributes to an overall stable protein. In cases of low level of sequence identity and/or few available amino acid sequences, however, the consensus residue of a specific position cannot be found easily or not at all. The structure-guided consensus approach utilizes structural information in addition to the frequency of an amino acid in a specific position of the sequence to point out residues for stabilization but then to reduce the number of residues in a given protein to be mutated and checked for stabilization.[2-3]

## A IV Homology model of all three generations of $CHMO_{Acineto}$

The structures were visualized by PyMOL[4] using first-ever crystalized $CHMO_{Acineto}$[5], which is a mutated variant as the template. The mutations introduced in this study are colored in red. The FAD is in yellow color.

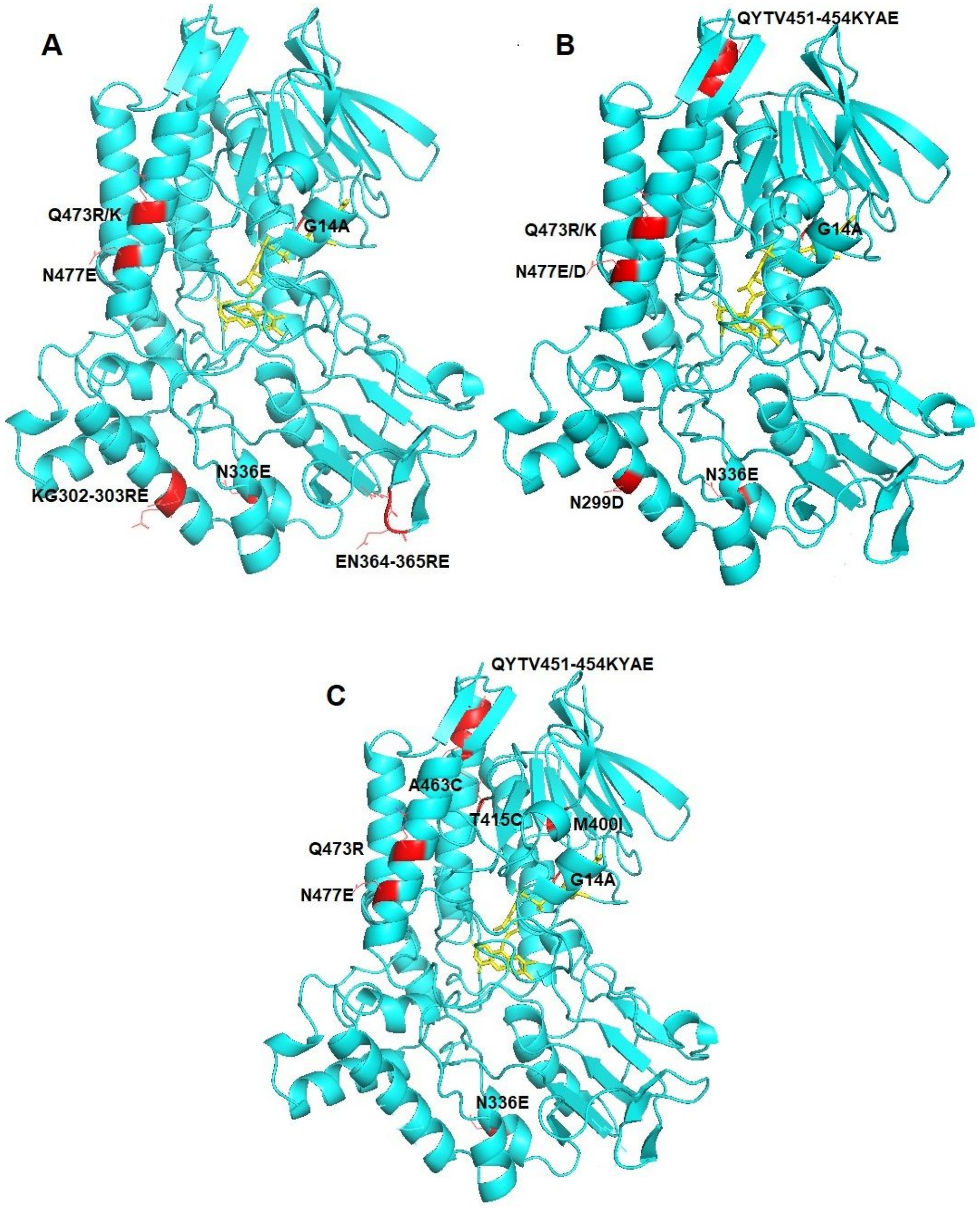


**Figure S3.** A) first generation B) second generation C) third generation variants locations on a homology model of $CHMO_{Acineto}$. Homology models were made based on the crystal structure of the $CHMO_{Acineto}$ mutant[5] as the template. Pymol was used to visualize the structures.[4]

## A V Library of mutants

**Table S2.** List of all variants in libraries with the abbreviation and mutations involved.

| | Amino acid position of mutations | | | | | | | | | | | | | | | |
|---|---|---|---|---|---|---|---|---|---|---|---|---|---|---|---|---|
| | **14** | **299** | **302** | **303** | **336** | **364** | **365** | **400** | **415** | **451** | **452** | **453** | **454** | **463** | **473** | **477** |
| **wt** | G | N | K | G | N | E | N | M | T | Q | Y | T | V | A | Q | N |
| **L1-1** | A | | | | | | | | | | | | | | | |
| **L1-2** | | | | | E | | | | | | | | | | | |
| **L1-3** | | | R | E | | | | | | | | | | | | |
| **L1-4** | | | | | | R | E | | | | | | | | | |
| **L1-5** | | | | | | | | | | | | | | | R | E |
| **L1-6** | | | | | | | | | | | | | | | K | E |
| **L1-7** | | | | | | | | | | K | Y | A | E | | | |
| **L2-1** | A | | | | | | | | | | | | | | R | E |
| **L2-2** | A | D | | | | | | | | | | | | | K | D |
| **L2-3** | A | D | | | | | | | | | | | | | R | D |
| **L2-4** | A | | | | | | | | | K | Y | A | E | | R | E |
| **L2-5** | A | | | | E | | | | | K | Y | A | E | | R | E |
| **L3-1** | A | | | | | | | | C | K | Y | A | E | C | R | E |
| **L3-2** | A | | | | | | | I | | K | Y | A | E | | R | E |
| **L3-3** | A | | | | E | | | I | C | K | Y | A | E | C | R | E |
| **L3-4** | A | | | | E | | | I | | K | Y | A | E | | R | E |
| **L3-5** | A | | | | E | | | I | C | K | Y | A | E | C | R | E |

## A VI Characterization of variants

**Table S3.** Characteristic of all variants. Kinetic stability was measured by incubation of 1 µM isolated enzyme at 30°C in 50 mM TrisHCl containing 10 µM FAD, pH 8.5. Thermodynamic stability was measured by nano differential scanning fluorimetry (nanoDSF) using 2 mg $mL^{-1}$ enzyme in 50 mM TrisHCl, 10 µM FAD, pH 8.5. Enzyme activity was measured by monitoring the decrease of NADPH absorbance at 340 nm. The activity assay mixture contained 0.05 µM CHMO, 100 µM NADPH, 0.5 mM cyclohexanone in 50mM TrisHCl pH 8.5 at 30°C. TTN values were obtained from the exponential fit of catalytic enzyme activity under turnover conditions [6].

| Variant | Activity (U/mg) | Ratio (to wt) | $T_m$ (℃) | Ratio (to wt) | Half-life (min) | Ratio (to wt) | TTN(*$10^4$) | Ratio (to wt) |
|---|---|---|---|---|---|---|---|---|
| wt | 16.40±1.1 | 1.00 | 38.2 | 1.00 | 34.4±4.6 | 1.00 | 5.07 | 1.00 |
| L1-1 | 18.50±1.60 | 1.13 | 39.6 | 1.04 | 44.1±4.7 | 1.28 | 7.32 | 1.44 |
| L1-2 | 22.80±4.90 | 1.39 | 38.7 | 1.01 | 34.8±3.8 | 1.01 | 7.11 | 1.40 |
| L1-3 | 18.02±1.30 | 1.10 | 37.8 | 0.99 | 22.0±9.5 | 0.64 | 3.54 | 0.70 |
| L1-4 | 17.28±2.20 | 1.05 | 38.0 | 0.99 | 32.5±12.5 | 0.94 | 5.03 | 0.99 |
| L1-5 | 20.02±4.60 | 1.22 | 39.4 | 1.03 | 27.2±8.0 | 0.79 | 4.78 | 0.94 |
| L1-6 | 08.07±1.60 | 0.49 | 38.9 | 1.02 | 30.6±2.5 | 0.89 | 2.21 | 0.44 |
| L1-7 | 21.90±3.10 | 1.34 | 39.1 | 1.02 | 41.2±5.5 | 1.20 | 8.08 | 1.59 |
| L2-1 | 19.30±1.25 | 1.18 | 40.2 | 1.05 | 47.0±2.1 | 1.37 | 8.12 | 1.60 |
| L2-2 | 15.40±2.20 | 0.94 | 38.2 | 1.00 | 26.0±3.0 | 0.76 | 3.59 | 0.71 |
| L2-3 | 17.60±2.01 | 1.07 | 38.1 | 1.00 | 24.6±1.0 | 0.72 | 3.88 | 0.77 |
| L2-4 | 22.60±1.06 | 1.38 | 40.1 | 1.05 | 92.3±3.04 | 2.68 | 18.72 | 3.69 |
| L2-5 | 23.6±4.20 | 1.44 | 40.9 | 1.07 | 97.7±10.8 | 2.84 | 20.67 | 4.08 |
| L3-1 | 24.7±3.20 | 1.51 | 41.4 | 1.08 | 79±2.4 | 2.30 | 17.46 | 3.44 |
| L3-2 | 16.7±1.70 | 1.02 | 42.6 | 1.12 | 97.1±15.4 | 2.82 | 14.57 | 2.87 |
| L3-3 | 25.5±4.03 | 1.55 | 42.0 | 1.10 | 181.1±20.7 | 5.26 | 41.38 | 8.16 |
| L3-4 | 17.4±1.60 | 1.06 | 42.5 | 1.11 | 275.6±75.9 | 8.01 | 42.9 | 8.46 |
| L3-5 | 17.5±2.10 | 1.07 | 42.7 | 1.12 | 130.7±24.3 | 3.80 | 20.44 | 4.03 |

**Table S4.** Kinetic measurements. Catalytic rates ($K_m$, $k_{cat}$) observed upon incubation of the isolated enzyme with varying amounts of the substrate (cyclohexanone) fitted to the Michaelis-Menten equation. The Kd was determined by fitting the data of catalytic activity of the holoenzyme versus concentration of FAD with a logistic function (Origin 8.5 for Windows) [7].

| Variant | $K_m$ (µM) | Ratio (to wt) | $k_{cat}$ ($s^{-1}$) | Ratio (to wt) | $k_{cat}/K_m$ ($mM^{-1} s^{-1}$) | Ratio (to wt) | Kd (µM) | Ratio (to wt) |
|---|---|---|---|---|---|---|---|---|
| wt | 6.7±2 | 1 | 15±1.3 | 1 | 2220 | 1 | 1.6±0.06 | 1 |
| L1-1 | 3.5±0.3 | 0.52 | 24.2±1.4 | 1.6 | 7058 | 3.17 | 0.19±0.07 | 0.1 |
| L2-5 | 3.97±0.7 | 0.59 | 13.8±1.5 | 0.92 | 3499 | 1.5 | | |
| L3-1 | 8.53±0.15 | 1.2 | 18.7±2.3 | 1.24 | 2191 | 0.98 | | |
| L3-2 | 3.37±0.07 | 0.49 | 11.8±1.5 | 0.78 | 3527 | 1.5 | | |
| L3-3 | 4.6±0.4 | 0.68 | 17.9±5.4 | 1.19 | 3902 | 1.7 | | |
| L3-4 | 5±1.5 | 0.74 | 16.3±1.8 | 1.08 | 3187 | 1.4 | 0.14±0.02 | 0.08 |
| L3-5 | 4.5±1.4 | 0.67 | 13.7±1.7 | 0.91 | 2941 | 1.3 | | |

## A VI.1 Evaluation of melting temperature ($T_m$)

Data analysis has been performed using NT Melting Control software (NanoTemper Technologies GmbH). The melting temperature ($T_m$) was determined by fitting the tryptophan fluorescence emission ratio of 350 nm to 330 nm using a polynomial function, in which the maximum slope is indicated by the peak of its first derivative.

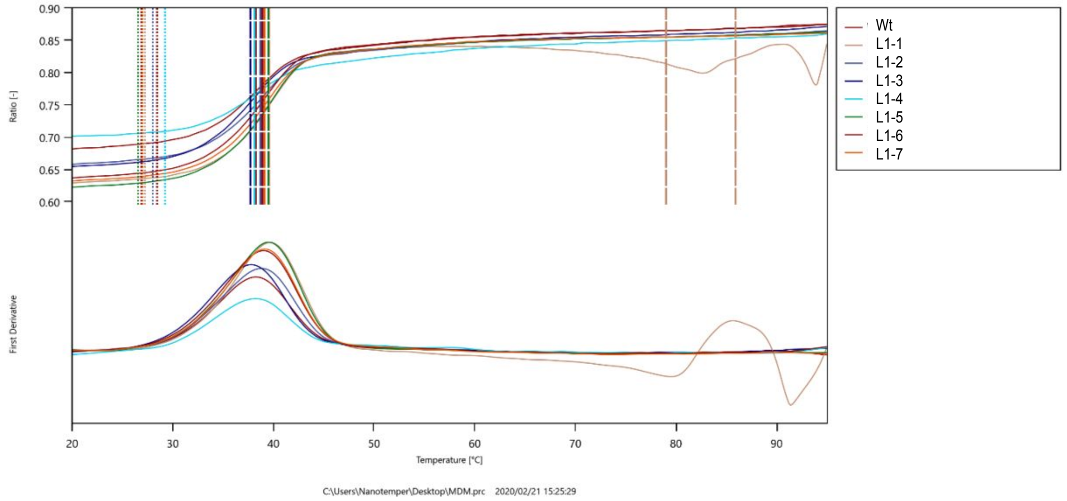


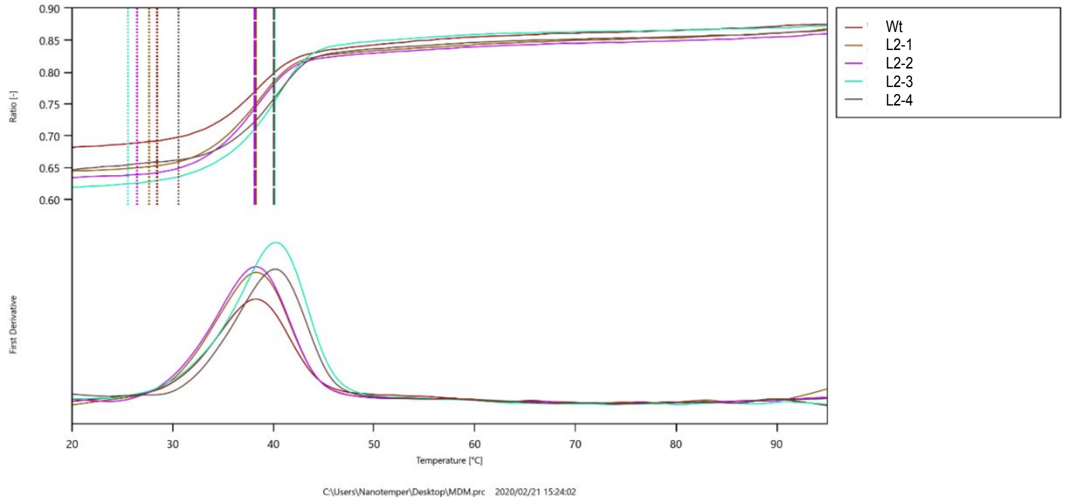

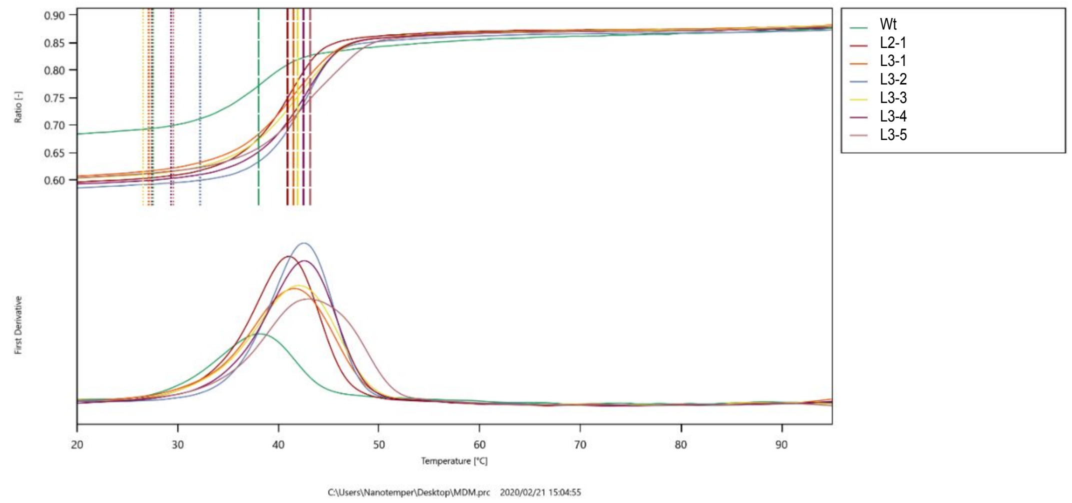


**Figure S4.** Melting temperature diagram for all the variants. The samples were prepared in TrisHCl 50 mM pH 8.5, 10 µM FAD with a final enzyme concentration of 2 mg $mL^{-1}$ and the samples run from 20 to 95°C..[8]

## A VII Characterization of site saturated G14 position

Site saturation mutagenesis was carried out by using the Q5 site directed mutagenesis kit. This was performed to evaluate the effect of different amino acids in position 14. The primers were designed using the NEBbaseChanger (https://nebasechanger.neb.com).

**Table S5.** Characterization of variants from site saturation mutagenesis. Thermodynamic stability measured by nano differential scanning fluorimetry (nanoDSF): 50 mM TrisHCl, 10 μM FAD, 2 mg $mL^{-1}$ enzyme. Enzyme activity was measured by monitoring the decrease of NADPH absorbance at 340 nm. Standard assays contained the enzyme (0.05 μM), cyclohexanone (0.5 mM) and NADPH (100 μM) in 50 mM TrisHCl pH 8.5. The product was extracted with ethyl acetate containing 0.1 mM methyl benzoate as the internal standard for the GC analysis. 4-methylcyclohexanone was used as the substrate for biotransformation.

| Variant | $T_m$ (°C) | Activity (U/mg) | GC (relative conversion %) |
|---|---|---|---|
| WT | 38.9 | 16.4±1.1 | >99 |
| G14A | 39.8 | 18.5±1.6 | 92 |
| G14L | n.a* | n.a | n.a |
| G14F | n.a | n.a | n.a |
| G14Y | 28.3 | n.a | 5.5 |
| G14H | n.a | n.a | n.a |
| G14N | 30.6 | n.a | 24 |
| G14Q | n.a | n.a | n.a |
| G14I | 25 | n.a | <3 |
| G14T | 27.6 | n.a | 5.6 |
| G14E | n.a | n.a | n.a |
| G14R | 39.2 | 11.5±0.3 | 94 |
| G14W | 28.9 | n.a | 13.7 |
| G14C | n.a | n.a | n.a |
| G14D | 23 | n.a | 30 |
| G14P | n.a | n.a | n.a |
| G14K | n.a | n.a | n.a |

n.a= not applicable

## A VII.1 **Circular dichroism (CD) spectroscopy**

CD spectroscopy is a useful technique to investigate the secondary structure and folding properties of proteins [9]. This technique is helping to determine if the purified protein is folded, or if a mutation affects its conformation. As it is shown in Figure S5, the only mutation that affected enzyme structure is the substitution of glycine with cysteine (G14C). This means that the reason why the variants are not active is not due to unfolding. The variants are either fragile, and thus can't not survive the purification process and lose their activity, or have no ability anymore to bind to FAD to perform the reaction.

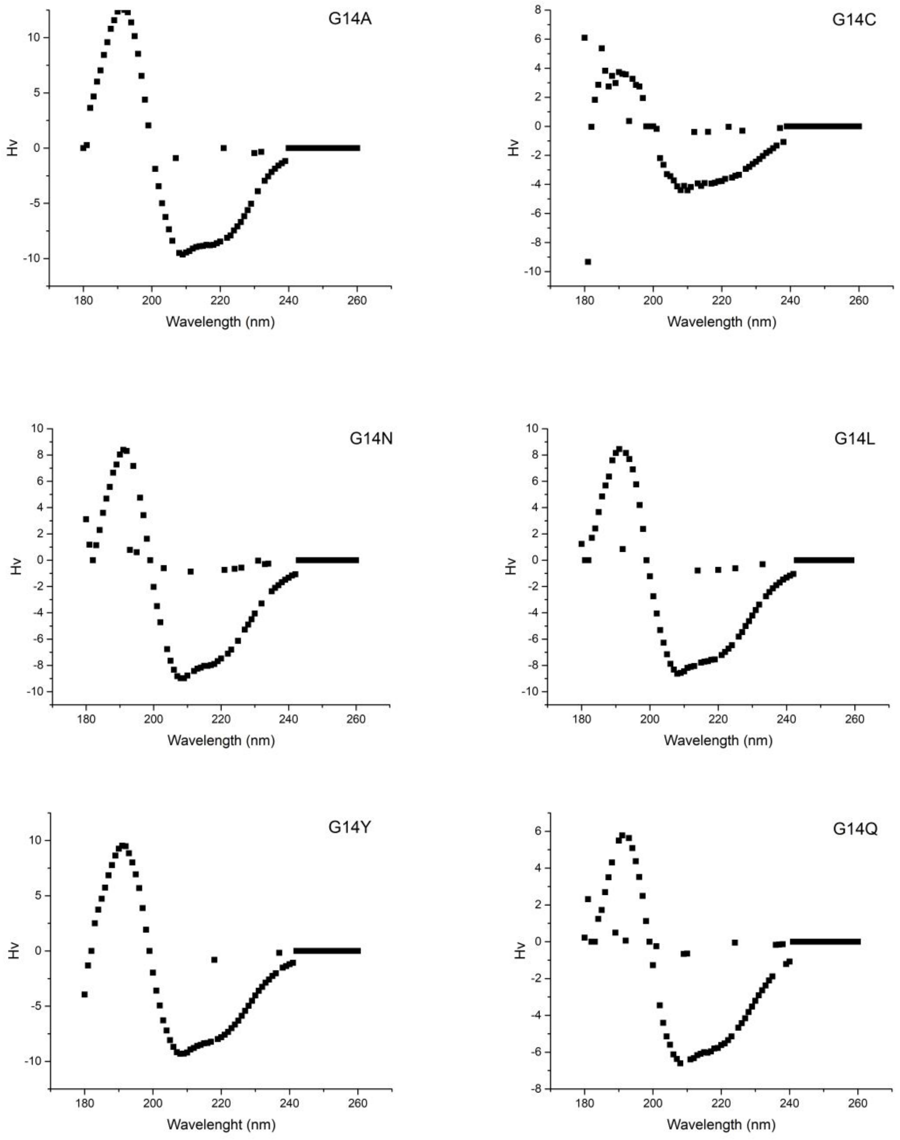

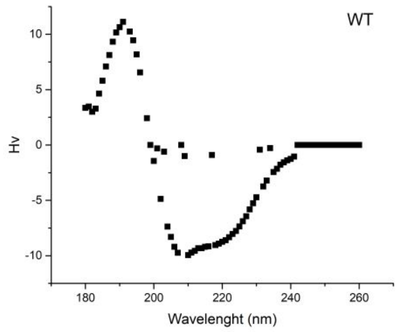


**Figure S5.** CD spectroscopy measurements of site saturated variants of L1-1. The isolated enzyme was diluted into distilled water with a final concentration of 0.1 mg $mL^{-1}$.

# A VIII Substrate profile

The substrate scope and the enantioselectivity of a few variants were evaluated to determine if the mutations have changed the selectivity of variants or not. The reactions were performed overnight at 30℃ in a shaking incubator operating at 200 rpm (Table S6 & Figure S7).

**Table S6.** Substrate profile of variants. The product was extracted with ethyl acetate containing 0.1 mM methyl benzoate as the internal standard for the GC analysis. The reference compound was cyclohexanone. This was used for control biotransformation to make sure the viability of purified enzymes.

| Substrate | 1 | | 2 | | 3 | | 4 | | 5 | |
|---|---|---|---|---|---|---|---|---|---|---|
| | Con[A] | ee[B] | Con | ee | Con | ee | Con | ee | Con | ee |
| WT | +++[C] | 99(S) | +++ | 99 (4S,6R) | + | 95 (R) | + | 76 (S) | +++ | N:ABN[D] 51:49 95(-):99(-) |
| L2-5 | +++ | 99(S) | +++ | 99 (4S,6R) | + | 95 (R) | + | 72 (S) | +++ | N:ABN 51:49 95(-):99(-) |
| L3-1 | +++ | 99(S) | +++ | 99 (4S,6R) | + | 95 (R) | + | 76 (S) | +++ | N:ABN 51:49 95(-):99(-) |
| L3-2 | +++ | 99(S) | ++ | 99 (4S,6R) | + | 95 (R) | + | 80 (S) | +++ | N:ABN 51:49 95(-):99(-) |
| L3-3 | +++ | 99 (S) | ++ | 99 (4S,6R) | + | 95 (R) | + | 49 (S) | +++ | N:ABN 51:49 95(-):99(-) |
| L3-4 | +++ | 99(S) | ++ | 99 (4S,6R) | + | 95 (R) | + | 74 (S) | +++ | N:ABN 51:49 95(-):99(-) |
| L3-5 | +++ | 99 (S) | ++ | 99 (4S,6R) | + | 95 (R) | + | 75 (S) | +++ | N:ABN 51:49 95(-):99(-) |

[A]Relative conversion (Conv) of the substrate to product

[B]Enantiomeric excess (*ee*) of product

[C] 10-50%: +, 50-80%: ++, 80-100%: +++

[D]N:ABN ratio of normal to abnormal lactone

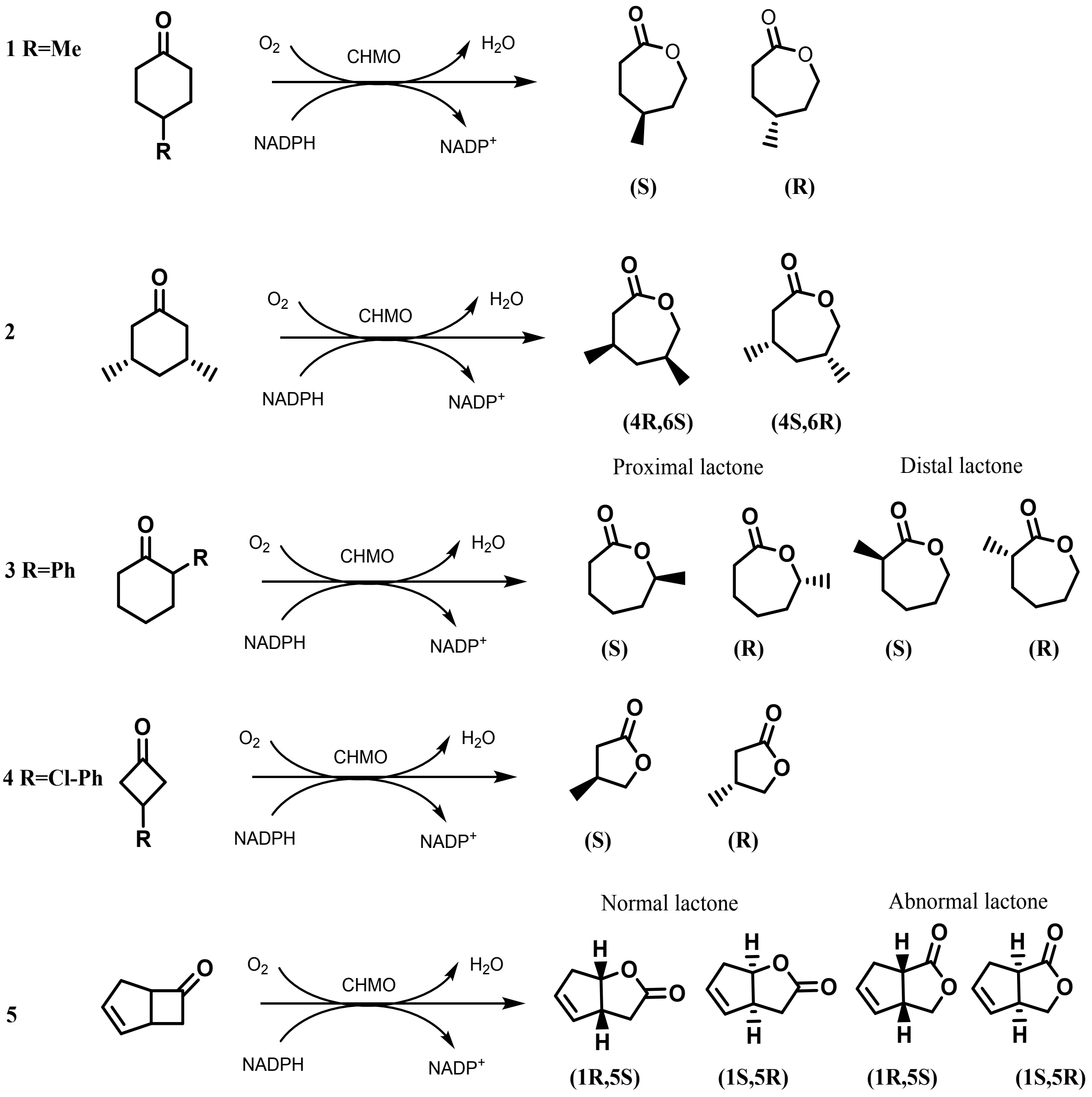


**Figure S6.** List of compounds used for the enantioselectivity evaluation.

## A IX **MD simulation and structure analysis**

The protonation states of the amino acids were assigned based on a pH 7 and oxidized form of FAD to reproduce the resting state of the enzyme. All the MD simulations were performed using the GROMOS simulation package (https:/www.gromos.net) [10], and the models were parameterized using the 54A8 GROMOS forcefield for both the protein and the FAD [11]. Short energy minimizations were performed using the steepest-descent algorithm in the vacuum. The models were then placed in a periodic rectangular water box with simple point charge (SPC) water molecules, leaving a minimum distance of 1.4 nm from the solute to the box walls. The systems were further minimized using the

steepest-descent algorithm with position restraints on the solute atoms. Counter ions were added by replacing water molecules with the most favorable electrostatic potential to reach charge neutralization using the program ion provided in the GROMOS++ package.[12] Five replicas for each model were generated by setting different initial velocities sampled from a Maxwell-Boltzman distribution at 60 K. The systems were thermalized up to 300 K by five discrete steps with position restraints on the solute atoms. The strength of the restraints was decreased by a factor 10 from 2.5 × 104 to 0 kJ mol−1nm−2. Production simulations of 50 ns each were performed at a constant temperature of 300 K and a constant pressure of 1 atm using the Nosé-Hoover-chains algorithm for the temperature control with 5 chains [13], and the weak coupling algorithm[14] for the pressure, with a coupling time of 0.5 ps and estimated isothermal compressibility of 4.575 x 10-4 $kJ^{-1}$ mol nm3. Newton's equations of motion were integrated using the leapfrog algorithm with a time step of 2 fs. The SHAKE algorithm [15] was used to maintain the bond lengths at their optimal values. Long-range electrostatic interactions beyond a cutoff of 1.4 nm were truncated and approximated by a generalized reaction field [16] with a relative dielectric permittivity of 61.[17] Nonbonded interactions up to a distance of 0.8 nm were computed at every time step using a pairlist that was updated every 10 fs. Interactions up to 1.4 nm were computed at pairlist updates and kept constant in between. The 5 first nanoseconds of the simulation were used as equilibration and discarded for the analysis. The root mean square deviations (RMSD) and root mean square fluctuations (RMSF) were computed for each simulation using the GROMOS++ package. The electrostatic potential study carried out using PyMOL[4].

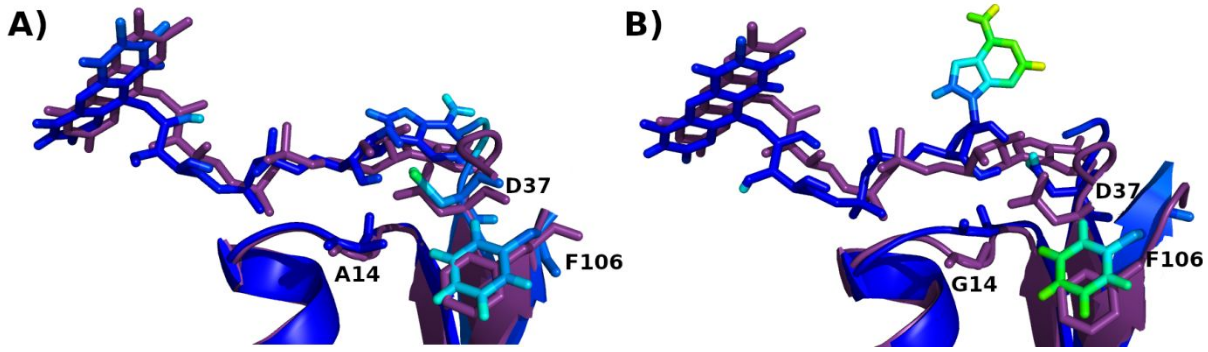


**Figure S7.** 3D representation of the average position of one of the simulations compared to the reference structure (purple) for the G14A mutant (A) and the wildtype (B). The color scale indicates the magnitude of the fluctuations, ranging from blue (low fluctuation).

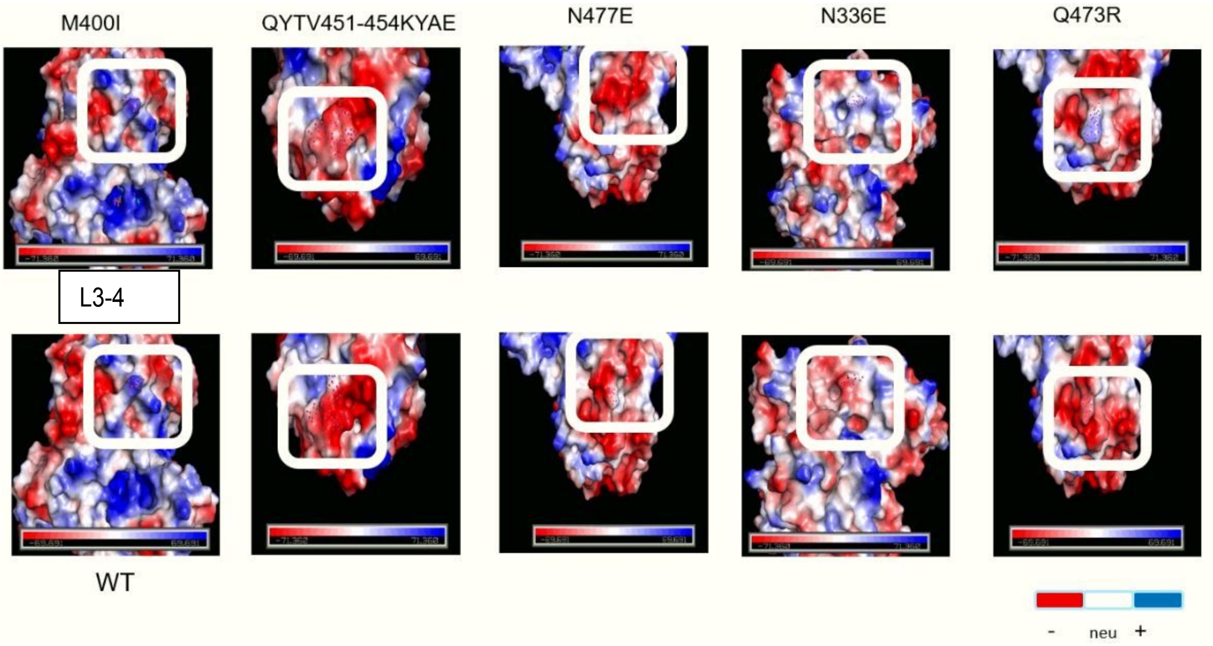


**Figure S8.** Comparison of the surface electrostatic potential of original amino acids and mutated amino acids of L3-4. The visualization and calculation were performed using PyMol.[4]

# B Materials and Methods

## B I Plasmid map and cloning plan

All the variants in this study were cloned in pET22 b (+) by using the NdeI, and NotI restriction sites, and all contained the His-Tag at C-terminal. The first and second series of mutants have been prepared by Dr. Saima Feroz [18] and the rest of mutants and new putative BVMOs synthesized by Genscript company with the same plan.

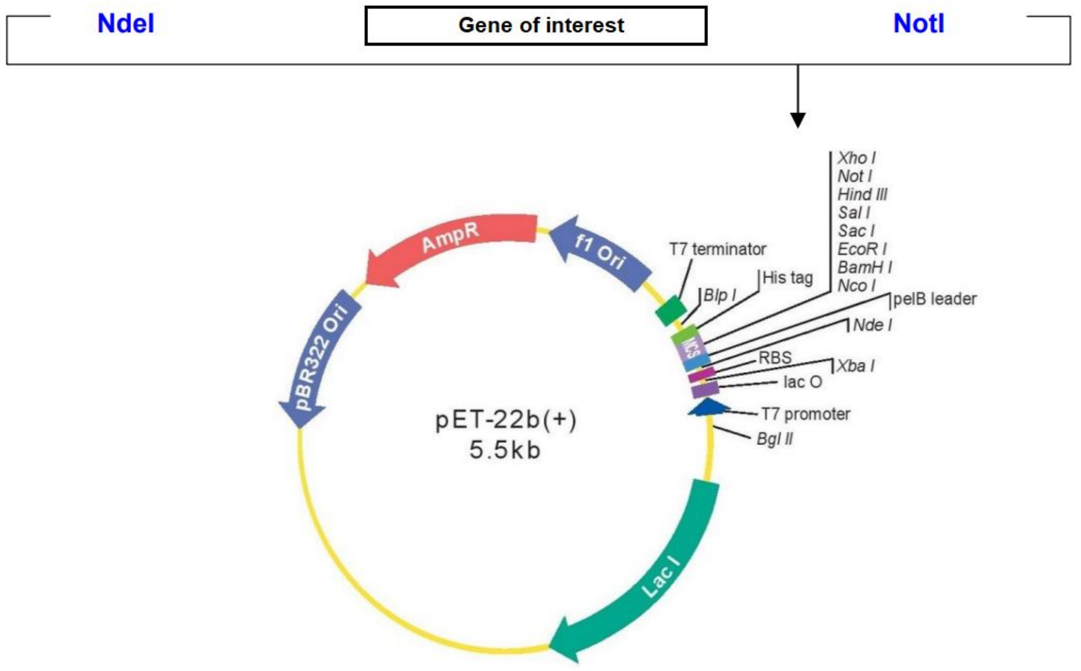


**Figure S9.** Vector map of pET22b(+), 17arbouring the gene of $CHMO_{Acineto}$ and all mutants.

## B II Site saturation mutagenesis

Site saturation mutagenesis of G14 residue was carried out using the Q5 site directed mutagenesis kit according to the protocol provided by the kit.

The mutations within the library ($1^{st}$-$3^{rd}$ generation) were introduced using the QuikChange® II XL Site-Directed Mutagenesis kit from Stratagene for the first and second libraries by following the procedure described by the manufacturer. The mutants in the third generation library were synthesized and cloned by Genscript.

## B III Enzyme expression and purification

*E. coli* strain BL21(DE3) was used for expression. LB medium (5 mL) supplemented with ampicillin (final concentration 100 µg/ml) was inoculated with *E. coli* BL21(DE3) pET22b(+)_$CHMO_{Acineto}$. Subsequently, the medium was incubated in an orbital shaker at 37 °C, 200 rpm overnight. Pre-cultivated bacteria (2 % v/v) were transferred to a 1 L flask containing 250 ml LB supplemented with 100 µg/ml ampicillin. The medium was incubated at 37 °C, 200 rpm for 2-3 hours to reach an optical density between 0.6-0.8 at 590 nm. Then, Isopropyl β-d-thiogalactopyranoside (IPTG) was added to the final concentration of 50 µM, and expression was carried out at 20 °C for 18-22 h.

All further steps were carried out at 4 ºC to protect the enzyme against inactivation. The culture containing expressed recombinant cells was centrifuged at 6000 rpm, 4 ℃ for 10 min, and cells were collected. Then, cell pellets were resuspended in 50 mM TrisHCl, pH 8 containing 100 µM FAD, and 100 µM of PMSF (phenylmethylsulfonyl fluoride). Afterward, the crude cell extract was sonicated by a Bandelin KE76 sonotrode connected to a Bandelin Sonoplus HD 3200 in 12 cycles (5s pulse, 55s break, amplitude 50 %). Cell debris and aggregates were removed by centrifugation (12000 rpm, 25 min, 4 ºC, JA-17 Beckmann rotor). The supernatant was filtered using a 0.25 µm filter, equilibrated with 50 mM TrisHCl, pH 8, 0.5 M NaCl, 100 µM FAD, and applied on 1 mL of Ni- sepharose column (1 mL, GE Healthcare Bioscience). The unwanted non-attached proteins were washed by using a 5 column volume of 50 mM TrisHCl, 0.5 M NaCl, 40 mM imidazole, 100 µM FAD, pH 8. The elution was achieved by applying 5 column-volumes of 50 mM TrisHCl, 0.5 M NaCl, pH 8.5 containing 400 mM imidazole, and 100 µM FAD. The eluted enzymes were washed by 50 mM TrisHCl, 100 µM FAD, pH 8.5, and concentrated with an ultracentrifugal tube with a cut-off of 10 kDa.[7]

## B IV Apoenzyme preparation (Deflavination)

All these procedures were performed at 4 °C to protect the enzyme from deactivation. The column chromatography method was used to generate CHMO FAD-free apoenzyme. Clear cell-free extract of the enzyme was prepared and equilibrated 1:1 with binding buffer (50 mM TrisHCl, 0.5 M NaCl, 25 mM imidazole, pH 8). The mixture was loaded at a flow rate of 1 mL min−1 into a Ni2+-Sepharose HP column (1 mL, GE Healthcare). Subsequently, the FAD was removed by washing the column with a 5-8 column volume of deflavination buffer (250 mM phosphate buffer, 3 M KBr, pH 8.0). The KBr weakens the bonding between the enzyme and FAD. Afterward, the column bound apoenzyme was eluted at a flow rate of 5 mL $min^{-1}$ with 5 column volume of elution buffer (50 mM TrisHCl, 0.5 M NaCl, 250 mM imidazole, pH 8). Finally, the apoenzyme was desalted with PD MiniTrap desalting column (PD MiniTrap G-25, GE Healthcare) using 50 mM TrisHCl, pH 8.5.).

## B V Enzyme activity and stability measurements

Enzyme activity was measured by monitoring the decrease of NADPH absorbance at 340 nm. Standard assays contained the enzyme (0.05 µM), cyclohexanone (0.5 mM), and NADPH (100 µM) in 50 mM TrisHCl, pH 8.5 at 30 °C [7]. The reaction was started immediately after enzyme addition by mixing 4 µL NADPH (25 mM stock solution) to the cuvette (final volume 1 mL). Oxidation of NADPH was followed at 30 °C in a Lambda 35 spectrophotometer (Perkin–Elmer, Waltham, MA, USA) for 120 seconds. To measure the kinetic stability (half-life), 10 µM enzyme in 50 mM TrisHCl, 10 µM FAD, pH 8.5 incubated at 30 °C. Samples were taken at different time points and added to a cuvette containing 100 µM NADPH and 0.5 mM substrate to measure the catalytic activity. The experimental data were fitted to an exponential decay equation using Origin Pro software (Origin 9.1 for Windows).

## B VI Kinetic measurements

**Michaelis constant** was measured to evaluate the affinity of variants toward the substrate (cyclohexanone). $K_m$ value was determined by measuring the activity in different concentrations of substrate (0-1000 µM). The $K_m$ value was measured by fitting the value of activity versus concentration of substrate with a Michaelis-Menten function.

**Dissociation constant (Kd) measurement:** Apoenzyme was prepared as described in supporting information. The catalytic activity of the isolated apoenzyme was determined to measure the dissociation constant (Kd). For this purpose, 1 µM apoenzyme was incubated with different quantities of FAD (0–100 µM, 5 min incubation at room temperature), and activity was measured as described above. The Kd was determined by fitting the data of activity versus concentration of FAD with a logistic function (Origin 8.5 for Windows).

## B VII Melting temperature ($T_m$)

The melting temperature ($T_m$) was measured by Prometheus NT.48. The samples were prepared in TrisHCl 50 mM pH 8.5, 10 µM FAD with a final enzyme concentration of 2 mg $mL^{-1}$. The glass capillaries were filled with 10 µL of the enzyme solution, and the samples ran from 20 to 95 °C. Data were analysed using NT Melting Control software (NanoTemper Technologies GmbH). The melting temperature (Tm) was determined by fitting the tryptophan fluorescence emission ratio of 350 nm to 330 nm using a polynomial function, in which the maximum slope is indicated by the peak of its first derivative.

## B VIII **Biotransformation**

The resting cell method was used to perform the biotransformation. Recombinant protein expression was done as described before. Cells were collected by centrifugation (6000 rpm, 4 °C, 10 min), resuspended and washed in 50 mM PBS buffer pH 7.4. After washing, the cells were centrifuged (6000 rpm, 4 °C, 10 min) and resuspended again with the same buffer to reach $OD_{590}$= 30. The reaction contained 1 mL ($OD_{590}$=30) of recombinant expressed cells suspended in PBS buffer (pH 7.4, 50 mM) and 10 mM substrate final concentration (methanol as cosolvent (5 % of total volume)). The components of the reaction (1.02 mL in total) were added into 25 mL flask, and the reaction was performed at 30 °C by shaking (220 rpm) for 24 h.[19] The product was extracted with ethyl acetate containing 0.1 mM methyl benzoate as the internal standard for the GC analysis. The product analysis was performed with GC (Thermo Scientific Trace or Focus GC, Thermo Fisher Scientific, Waltham, MA, USA) using the chiral/achiral column. Product validation was carried out by literature-known reference biotransformations.

# C References


(1) Edgar, R. C., Muscle: Multiple Sequence Alignment with High Accuracy and High Throughput. *Nucleic. Acids. Res.* **2004,** *32* (5), 1792-7.
(2) Vázquez-Figueroa , E.; Chaparro-Riggers , J.; Bommarius, A. S., Development of a Thermostable Glucose Dehydrogenase by a Structure-Guided Consensus Concept. *ChemBioChem* **2007,** *8* (18), 2295-2301.
(3) Amin, N.; Liu, A. D.; Ramer, S.; Aehle, W.; Meijer, D.; Metin, M.; Wong, S.; Gualfetti, P.; Schellenberger, V., Construction of Stabilized Proteins by Combinatorial Consensus Mutagenesis. *Protein Engineering, Design and Selection* **2004,** *17* (11), 787-793.
(4) DeLano, W. L., Pymol : An Open-Source Molecular Graphics Tool. *PyMOL.* **2002,** *DeLano Scientific, San Carlos, CA, 700.*
(5) Zhang, Y.; Wu, Y.-Q.; Xu, N.; Zhao, Q.; Yu, H.-L.; Xu, J.-H., Engineering of Cyclohexanone Monooxygenase for the Enantioselective Synthesis of (S)-Omeprazole. *ACS. Sustain. Chem. Eng.* **2019,** *7* (7), 7218-7226.
(6) Rogers, T. A.; Bommarius, A. S., Utilizing Simple Biochemical Measurements to Predict Lifetime Output of Biocatalysts in Continuous Isothermal Processes. *Chem. Eng. Sci.* **2010,** *65* (6), 2118-2124.
(7) Goncalves, L. C. P.; Kracher, D.; Milker, S.; Fink, M. J.; Rudroff, F.; Ludwig, R.; Bommarius, A. S., Mutagenesis-Independent Stabilization of Class B Flavin Monooxygenases in Operation. *Adv. Synth. Catal.* **2017,** *359*, 2121-2131.
(8) Mansouri, H. R.; Mihovilovic, M. D.; Rudroff, F., Investigation of a Novel Type I Baeyer-Villiger Monooxygenase from *Amycolatopsis Thermoflava* Revealed High Thermodynamic- but Limited Kinetic Stability. *ChemBioChem* **2019**.
(9) Greenfield, N. J., Using Circular Dichroism Spectra to Estimate Protein Secondary Structure. *Nat Protoc* **2006,** *1* (6), 2876-2890.
(10) Schmid, N.; Christ, C. D.; Christen, M.; Eichenberger, A. P.; van Gunsteren, W. F., Architecture, Implementation and Parallelisation of the Gromos Software for Biomolecular Simulation. *Comput. Phys. Commun.* **2012,** *183* (4), 890-903.
(11) Reif, M. M.; Hünenberger, P. H.; Oostenbrink, C., New Interaction Parameters for Charged Amino Acid Side Chains in the Gromos Force Field. *J. Chem. Theory Comput.* **2012,** *8* (10), 3705-3723.
(12) Eichenberger, A. P.; Allison, J. R.; Dolenc, J.; Geerke, D. P.; Horta, B. A. C.; Meier, K.; Oostenbrink, C.; Schmid, N.; Steiner, D.; Wang, D.; van Gunsteren, W. F., Gromos++ Software for the Analysis of Biomolecular Simulation Trajectories. *J. Chem. Theory Comput.* **2011,** *7* (10), 3379-3390.
(13) Martyna, G. J.; Klein, M. L.; Tuckerman, M., Nosé–Hoover Chains: The Canonical Ensemble Via Continuous Dynamics. *J. Chem. Phys.* **1992,** *97* (4), 2635-2643.
(14) Berendsen, H. J. C.; Postma, J. P. M.; van Gunsteren, W. F.; DiNola, A.; Haak, J. R., Molecular Dynamics with Coupling to an External Bath. *J. Chem. Phys.* **1984,** *81* (8), 3684-3690.
(15) Ryckaert, J.-P.; Ciccotti, G.; Berendsen, H. J. C., Numerical Integration of the Cartesian Equations of Motion of a System with Constraints: Molecular Dynamics of N-Alkanes. *J. Comput. Phys.* **1977,** *23* (3), 327-341.
(16) Tironi, I. G.; Sperb, R.; Smith, P. E.; van Gunsteren, W. F., A Generalized Reaction Field Method for Molecular Dynamics Simulations. *J. Chem. Phys.* **1995,** *102* (13), 5451-5459.
(17) Heinz, T. N.; van Gunsteren, W. F.; Hünenberger, P. H., Comparison of Four Methods to Compute the Dielectric Permittivity of Liquids from Molecular Dynamics Simulations. *J. Chem. Phys.* **2001,** *115* (3), 1125-1136.
(18) Feroz, S. Improving the Thermostability of Baeyer-Villiger Monooxygenases. TU Wien, Austria, 2012.

(19) de Gonzalo, G.; Fürst, M.; Fraaije, M., Polycyclic Ketone Monooxygenase (Pockemo): A Robust Biocatalyst for the Synthesis of Optically Active Sulfoxides. *Catalysts.* **2017,** *7* (10), 288.
